# A Therapeutic Arrangement and Syllabus of Materia Medica

**Published:** 1836-01

**Authors:** 


ART. VII.—*A Therapeutic Arrangement and Syllabus of Materia Medica.* By JAMES JOHNSTONE, M.D., Fellow of the College of Physicians, and Physician to the General Hospital, Birmingham. *London*, 1835. Small 8vo. pp. 84.

IN the first part of this work the substances used in medicine are arranged according to their therapeutic action; in the second part those derived from the animal and vegetable kingdoms are arranged respectively according to the systems of Cuvier and Jussieu, and the minerals are given in alphabetical order. A posological table is annexed, containing about 400 drugs or officinal preparations, with their doses, which is drawn up with remarkable accuracy. The following extracts may give our readers some notion of the manner of this succinct but comprehensive manual.

"CLASS IV. ORDER 1.—NARCOTICS.

| | |
|---|---|
| Aconitum. | Lauri baccæ. |
| Belladonna. | Lactucæ extractum. |
| Conium. | Lactucarium. |
| Humulus. | Opium. |
| Lupulina. | Morphia. |
| Hyoscyamus. | Narcotia. |
| Camphora. | Stramonium." p. 10. |

"SCROFULARIÆ. DIGITALIS PURPUREA.—THE PURPLE FOXGLOVE.
LINN. Class *Didynamia;* Order *Angiospermia.*

*Officinal.*—Digitalis folia, Foxglove leaves.

*Analysis.*—Extractive; green oil; *digitaline;* salts.

*Medicinal properties.*—Sedative and diuretic.

Digitalis is a narcotico-acrid poison. When this medicine has been taken for several days, even in moderate doses, it frequently excites nausea, vomiting, giddiness, want of sleep, sense of heat throughout the body, and all external objects assume a green appearance; general depression, sometimes salivation, diarrhœa, or convulsions. The pulse is always feeble, and frequently intermits. When a single large dose of digitalis has been given, vomiting, purging, depression of the pulse, dilatation of the pupils, faintness, cold sweats, swelling of the face, and convulsions or coma are usually the consequence.

*Post mortem appearances.*—The membranes of the brain injected with blood; the mucous coat of the stomach of a red colour." p. 35.

| | |
|---|---|
| "Pulvis aloes compositus | gr. v. to gr. xv. |
| Pulvis antimonialis | gr. j. to gr. vij. |
| Pulvis cinnamomi compositus | gr. v. to gr. x. |
| Pulvis contrajervæ compositus | gr. x. to ʒss. |
| Pulvis cornu usti cum opio | gr. v. to ℈ss. |
| Pulvis cretæ compositus | gr. xv. to ʒss. |
| Pulvis cretæ comp. cum opio | gr. x. ℈j. |
| Pulvis ipecacuanhæ compositus | gr. v. to gr. xv. |
| Pulvis kino compositus | gr. v. to ℈j. |
| Pulvis scammoniæ compositus | gr. v. to gr. xv. |
| Pulvis sennæ compositus | ℈j. to ʒj. |
| Pulvis tragacanthæ compositus | gr. x. to ʒss." p. 81. |

This book cannot but be particularly useful to those who intend to lecture or write upon the Materia Medica; as well as to the students for whose particular use it is prepared.

